# Building a culture of quality in maternal and newborn health: experience from the quality, equity and dignity network in Ethiopia

**DOI:** 10.1080/16549716.2024.2433576

**Published:** 2024-12-02

**Authors:** Anene Tesfa Berhanu, Asebe Amenu Tufa, Seblewengel Lemma, Geremew Gonfa, Theodros Getachew, Desalegn Bekele, Fitsum Kibret, Nehla Djellouli, Tim Colbourn, Tanya Marchant

**Affiliations:** a Health Systems and Reproductive Health Directorate, Ethiopian Public Health Institute, Addis Ababa, Ethiopia; b Department of Disease Control, London School of Hygiene & Tropical Medicine, London, UK; c Health Systems Quality Directorate, Ethiopian Ministry of Health, Addis Ababa, Ethiopia; d Institute for Global Health, University College London, London, UK

**Keywords:** QED network, quality culture, quality improvement, people-centered care, leadership, motivation, rewards, ownership

## Abstract

**Background:**

Building a culture of quality is vital for sustaining high-quality healthcare, emphasising shared values and continuous improvement. The Quality Equity and Dignity (QED) network was a global initiative working toward this objective, focusing on maternal and newborn health. This paper aims to describe how QED influenced five identified attributes of quality culture in Ethiopia: leadership, people-centered interventions, collaboration, rewards, and ownership towards building and sustaining a culture of quality in healthcare establishments.

**Methods:**

This qualitative study, conducted at two points six months apart, incorporated data from key informant interviews, observations, and document reviews. It included 18 national and 22 sub-national key informant interviews, seven facility observations, and one technical working group meeting observation. Data analysis was performed using NVivo 12 software, focusing on identified thematic areas related to quality culture.

**Result:**

Leadership was crucial for building a quality culture in Ethiopia, and the QED network strengthened government leadership structures, although leadership capacity and staff turnover were challenges. QED enhanced people-centered care and data tracking, but the added data focus burdened healthcare workers. Opportunities for collaboration and shared learning were facilitated, although not accessible to all actors. Motivation and rewards encouraged good performance, but addressing intrinsic behavioral change remained a challenge.

**Conclusion:**

Achieving high-quality healthcare involves more than tools and infrastructure; it requires a cultural shift with behavior change consistently demonstrated at various levels. The QED network faced challenges in building a culture of quality but serves as an exemplary initiative for other networks to learn from.

## Paper Context

- **Main findings**: The quality equity dignity network significantly improved leadership, people-centered care, collaboration, rewards, and ownership in Ethiopian healthcare, enhancing maternal and newborn health outcomes.
- **Added knowledge**: The study provides empirical evidence of the quality equity dignity network’s effectiveness in promoting a quality culture in low-resource healthcare settings, addressing a gap in the literature.
- **Global health impact for policy and action**: The findings advocate for sustainable policies that improve maternal and newborn health globally by presenting the experiences of the quality equity and dignity network, encouraging others to learn and replicate its successes.

## Background

Improving maternal and newborn health (MNH) outcomes is a key priority of the Sustainable Development Goals (SDGs) [1]. A high-quality health system with a focus on the quality of health care is crucial to this goal, encompassing adequate health system structures, the availability of skilled compassionate health workers, adequate provision of care that is supported by community engagement, and robust health information systems, and positive care experiences [2,3]. In recent years, quality improvement (QI) innovations that apply a systematic and coordinated approach to solving people-centered problems to bring about measurable improvements in healthcare quality have become popular [4].

One characteristic of quality improvement is for healthcare providers to adopt continuous small steps towards behavioral change activities that eventually become a habit over time [5]. These individual-level changes sum up to bring an institutional environment conducive to better quality, which in turn grows into a culture of quality [6,7].

Quality culture is defined as an environment in which employees not only follow quality guidelines but also consistently see others taking quality-focused actions, hear others talking about quality, and feel quality all around them. It describes a system of shared values, beliefs, and norms that focus on continuous improvement of the quality of service provided. To succeed, a quality culture should be deeply embedded within all aspects of an organization so that it is seen as a way of working for all employees [5]. Establishing a culture of quality care in maternal and newborn health requires a multifaceted approach within the health system [3,7]. A global initiative working toward this objective has been the Quality, Equity, and Dignity (QED) network. This 11-country network aimed to halve maternal and newborn deaths and stillbirths in selected learning facilities and to improve clients’ experience of care [8,9]. It operated through quality improvement collaboratives, shared learning, and establishing a culture of quality at district and facility levels of network countries [10]. Ethiopia has been a QED network member country since 2017. The aims of the QED network were complemented by the government’s commitments to improve maternal and newborn health outcomes through the national healthcare quality and safety strategy which proposes to ‘create a quality culture through continuous learning and improvement’ as the fifth objective [11,12].

Relatively little has been reported about experiences of cultivating a sustained culture of quality health care, particularly in low-resource countries [13]. This manuscript investigates whether and how five attributes that help to build and sustain a culture of quality – leadership, people-centered interventions, collaboration, rewards, and ownership – were experienced by actors at the local level during the QED initiative in Ethiopia.

## Materials and methods

### Study setting

Ethiopia, with 117 million people, is Africa’s second most populous country after Nigeria, having a population growth rate of 2.5% [14]. The Ethiopian health system follows a hierarchical structure comprising the Federal Ministry of Health (FMOH) at the national level, which formulates policies and regulations. Regional Health Bureaus oversee healthcare delivery within each administrative region, operating under the FMOH’s guidance. At the district level (woredas), health offices manage healthcare services, while health facilities, including hospitals, health centers, and posts, provide care to communities. This tiered system ensures healthcare access from national to local levels, catering to diverse population needs. Beyond healthcare access, the FMOH includes a Quality Improvement Directorate, which is responsible for setting standards and monitoring healthcare quality through initiatives like the Maternal and Perinatal Death Surveillance and Response (MPDSR) system. At the regional level, Regional Health Bureaus adopt national policies to local needs, ensuring regional hospitals follow guidelines. The district health offices manage primary healthcare, monitor local health centers, and ensure compliance with national standards, particularly through the Health Extension Program.

In 2019 only half of births were attended by trained medical birth attendants [15]. Although progress has been observed, Ethiopia continues to have one of the highest maternal mortality ratios in the world, estimated to be 412 per 100,000 live births; and persistently high neonatal and under 5 mortality rates, estimated at 27.1 and 46.2 per 1,000 in 2022 respectively [15]. Similarly, in 2021, Ethiopia’s stillbirth rate was reported at 20.6 per 1,000 live births, a significant improvement from 32.6 per 1,000 live births in 2000. The stillbirth rate has been linked to complications during labor and inadequate quality of care in health facilities, but the decline reflects progress in healthcare services over two decades [16].

#### Quality equity and dignity network in Ethiopia

The QED network approach to fostering a quality culture was underpinned by the LALA framework (Leadership, Action, Learning, and Accountability), emphasizing the importance of leadership commitment, taking action on quality improvement, continuous learning and adaptation, and accountability for outcomes [17].

In Ethiopia, the QED network was implemented in 41 government learning facilities which were chosen from 12 learning districts spanning eight regions namely Afar, Amhara, Benshangul Gumuz, Gambella, Oromia, Sidama Southern Nations, Nationalities, and Peoples’ Region (SNNPR), Southwest Ethiopia, and one municipal administration Addis Ababa. Multiple organizations worked alongside the government to promote QED goals, including WHO, the Clinton Health Access Initiative, the Institute for Healthcare Care Improvement, and USAID to transform primary health care and health in developing regions [7,10].

The selected facilities were networked via learning platforms conducted at the facility, district, and national levels. Learning sessions were used to share exemplary and outstanding quality improvement projects, innovations, and lessons learned from facilities. Data of selected key indicators from these facilities were also collected and followed regularly with the help of partner organizations and regional health offices in some cases.

### Study design

A qualitative study comprising key informant interviews, non-participant observations, and document review was conducted to address pre-defined research questions. The primary emphasis of these questions lay on network emergence, legitimacy, and effectiveness [7,10,17,18]. This paper is a secondary analysis to evaluate the contribution of the network towards fostering a quality culture within institutions in Ethiopia. Data were collected at two points of time, approximately six months apart, from Jan 2021– Mar 2020 to Nov 2021- Dec 2021.

**Figure 1.**
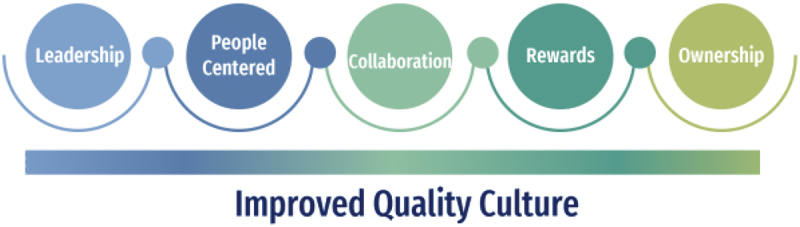
An adapted analytical framework depicting characteristics that foster a quality culture [4,8,18,19].

We based our investigation on an analytical framework (Figure 1) adapted from the literature on quality culture that has emerged from different fields of study [5,19,20]. Five key steps to fostering institutional quality culture are depicted.

The analytical framework builds from multi-sectoral learning to define five key attributes [4,8,19,20]. Leadership is crucial in establishing a robust framework for quality governance, while also demonstrating a commitment to quality, thereby inspiring health workers [8]. People-centered approaches necessitate continual reflection on the needs of health service users, while [6] collaboration is essential for promoting teamwork and shared learning across the organization [19]. Additionally, rewards and incentives for success motivate health workers to strive for continuous improvement, as is a sense of ownership of the services provided by health workers to foster involvement and empowerment, encourage honest communication, and generate innovative solutions to fact-based problems [20].

#### Key informant interviews

Topic guides were developed and piloted, tailored to respondents at the national and local levels. Both guides were translated into the local languages Amharic and Afan Oromo as necessary. QED network focal persons at each implementation partner office, national and regional health offices as well as providers in the member facilities participated in the study. Emerging issues in the first round of the study were further assessed in the follow-up round of data collection to fill any data gap identified. Participants were identified in consultation with project implementers and senior experts in the technical working group established at the national level. The assessment included network members acting as implementers and supporting the selected health facilities and health care providers in the facilities. Only those with at least six months of network membership/participation were included. Accordingly, 18 key informant interviews at the national and 22 at the sub-national levels were conducted.

#### Non-participant observations

Non-participant observations were conducted at the national technical working group meeting and two best and two least-performing QED network facilities were identified based on their national neonatal mortality rate, maternal mortality, and stillbirth data. The observations were conducted using a checklist developed to address the aims of the study, adopting the WHO checklist of the 15 common core indicators of QED [2,8]. Quality unit representatives of each observed health facility were also interviewed to supplement the data obtained from the observation. Seven facility observations and one QED network technical working group meeting observation were conducted over two rounds of data collection.

#### Document review

Global and local MNH-related policies, operational guidelines, and strategic documents related to the implementation of the QED network in Ethiopia were reviewed thoroughly. In addition, the facility-level hard copies of periodic meeting minutes and reports were included in our review.

### Data analysis

The recorded qualitative data were transcribed verbatim and coded with the qualitative software NVivo 12. We employed a coding framework derived from relevant theories to analyze qualitative data from interviews, observations, and document reviews. All interview transcriptions and observation notes were uploaded to NVivo for coding. Following coding under the identified thematic areas, the findings were triangulated with those from meeting notes and document reviews. The primary framework included ‘theory’ codes reflecting underlying theories, supplemented by ‘case study’ codes to distinguish data relevant to each case study. The codebook for this secondary analysis was prepared using a combination of inductive and deductive approaches, allowing for a comprehensive analysis of the data. We prepared a codebook to manage excerpts from respondents that pertained to the concept of quality culture, taking into account the five key attributes that have been identified in the analytical framework (Figure 1). This approach ensured that we captured a holistic understanding of how these attributes manifested in the context of healthcare quality culture.

In addition, emerging ideas from the first transcripts were also inductively integrated. The codebook was later validated by conducting an intercoder agreement test using sample transcripts and discussions. Following the data reduction and synthesis, thematic analysis was carried out for each thematic area identified.

### Ethical considerations

Ethical approval was obtained from the ethical review board of the Ethiopian Public Health Institute (EPHI-IRB-240-2020), London School of Hygiene and Tropical Medicine (ref 17,541), and University College London (3433/003). Before participating in the interview, each participant signed an informed written consent form.

## Results

The following section explores and organizes findings about the contributions of the QED Network to promote quality culture, based on the five major thematic areas identified: leadership emphasis, people-centered care, collaboration, motivation, and ownership.

### Leadership emphasis

This section will delve into the multifaceted dimensions through which effective leadership contributes to the establishment and perpetuation of a robust quality culture within healthcare systems. According to the study findings, strong leadership at the national and sub-national levels was essential in establishing a quality culture, along with the provision of adequate resources, the creation of institutional structures, commitment to putting strategies into practice, and other factors. In Ethiopia, the current national focus on healthcare quality from the government and partners’ side, as well as strategies that stipulate a shift from adequate health service coverage to quality of services, have provided a significant opportunity and leadership commitment to practice quality-improving activities.

The key factor here is leadership, from the CEO to the medical director, who actively encourages and suggests Quality Improvement projects based on identified gaps. They engage in our telegram communication groups, where we share daily information about neonatal mortality causes and admissions. This leadership involvement fosters a culture of problem-solving and interaction throughout the organization, promoting effective solutions from top to bottom. Interview-Round2-Local-Hospital.

According to study participants, the establishment of regional and facility-level quality structures, coupled with the appointment of quality focal persons, demonstrated a heightened level of commitment towards ensuring the delivery of high-quality services. The allocation of dedicated personnel to oversee the quality of services provided at various levels of the healthcare system was a clear indication that quality was not viewed as an afterthought, but rather as a core component of service delivery. Such initiatives not only provided a means for monitoring and evaluating the quality of services delivered but also ensured that quality improvement efforts were prioritized and systematically implemented. These efforts were built from existing government commitments, as described by a respondent from the Federal Ministry of Health, who attested to the significant progress made in improving the quality of healthcare services at the facility and regional levels.

I would like to point out that we have had the Ethiopian quality management structure since 2015 and this is a good thing. If you see the ministry, there is a health service quality directorate. Even, if you go to regions, they have an independent quality unit. Hospitals also have either a quality directorate or a quality focal person. At the health centers, they at least have a committee. I believe the presence of all these structures is the main facilitator. Interview-Round1-National-MOH

However, respondents stated that the leadership structure also needed capacity-building support. According to one of the regional health offices, many leaders were unaware of the system-building component of quality improvement, which entailed embedding and sustaining a quality culture in the institutions involved.

The capacity of healthcare leaders to deliver sustainable, quality care is lacking, as they have insufficient awareness of the importance of quality care and their role in achieving it. I’ve observed a significant challenge where leaders do not recognize the importance of quality care as part of the system-building process, and there’s a need for ongoing awareness-raising activities to address this issue. Interview_Round2_Local_RHB

QED network played a role in enhancing this leadership by introducing the LALA (Leadership, Action, Learning, and Accountability) framework within existing structures. The LALA framework approach has recently gained popularity for bringing multilevel and integrated quality improvement to this line.

If you ask me what value is added when this [the network] comes, as I told you, the introduction is new in terms of involvement and quality. Because to perform activities on quality, it is a must to have structure, like the LALA framework. The so-called leadership, learning, action including the new standard set has its contribution to performing clinical activities. Interview-Round1-National-MoH

However, QED plans for capacity building were negatively affected by the high staff turnover rate and limited staff. Some respondents indicated that a shortage of personnel limited activities at regional quality units:

The Regional quality structure is very weak and it’s understaffed. Even those assigned people have lots of responsibilities. They are assigned for every quality-related activity, not just for maternal and newborn health. Interview-Round2-National-Partner

### People-centered care

In this section, an elucidation of the relationship between people-centered care and the cultivation of a quality culture will be presented based on the experiences of the QED network. Our study participants described the network as a platform for understanding the needs of beneficiaries. The approach involves creating specific quality improvement projects and enhancing the capacity of healthcare providers to address and rectify identified gaps.

Now what matters is not only the performance but whether these mothers get quality services or not. This is their main task. If a gap is found on that, they design a quality improvement project based on that gap and capacitate themselves within that. Interview-Round1-Local-RHB

Respondents highlighted the long-term impact of patient-oriented care, noting practical improvements in institutional delivery and the health-seeking behaviors of beneficiaries. The ‘quality equity and dignity’ approach, which centers on a respectful healthcare delivery system, is particularly valued for its positive influence on the health-seeking behaviors of respondents.

… We are seeing outcomes due to respectful maternal care. We are seeing an increase in the number of institutional deliveries per day. At [X] hospital about 20 mothers deliver per day. So, I can say there is a huge motivation for the health care service, and the respectful maternity care has brought such changes. Interview-Round2-Local-RHB

Participants noted that the QED network revitalized previously overlooked routine patient care monitoring systems, such as partograph registration, infection prevention, and counseling. On-the-job training, along with technical and material support, has empowered healthcare providers to exercise their expertise without limitations.

For example, as a midwife I was supposed to fill a partograph, write history and prepare a summary note, to say that I have conducted a delivery. With the current health centre’s manpower and workload, it is difficult to perform all these tasks. However, now we understand the service will not be complete without such things and we know that it is our duty. But it has created a burden … . there are workloads added such as filling partographs and performing audits every week. We perform audits on Sunday evenings or Monday early in the morning. Interview-Round1-Local-Health Center

At the national level, a considerable effort has been made to reduce institutional mortality by widely establishing active and responsive clinical audits and the MPDSR (Maternal and Perinatal Death Surveillance and Response) system. In line with this, the QED network aimed to improve client satisfaction and introduced a monitoring system with 15 client-centred common core indicators, categorized into three subthemes: health care service provision, water, sanitation and hygiene and experience of care.

…It also measures the experience of care that we didn’t measure before, and it brings a new perspective. Interview-Round1-National-MOH

Most respondents agree with the importance of the ‘experience of care component’. Despite the ambition to consistently monitor satisfaction levels and enhance people-centered care, obtaining truthful and reliable data from mothers who visited facilities proved challenging, given the difficulties in ensuring unbiased data collection.

I think the most important thing is the experience of care. If the people at the site level worked well on this, we would know the level of satisfaction. But it was not done. The first reason is it is a subjective component. How can a doctor ask a person he treated, about the experience of care? If an independent person is to do it, there must be an additional resource. So it has created a problem. But it is one of the most important indicators of quality. Interview-Round1-National-MOH

Overall, QED has motivated healthcare workers to apply their existing knowledge in providing quality care, but assessing to what extent it contributed remains challenging.

### Collaboration

Collaboration includes learning and sharing experiences across healthcare workers and facilities, encouraging peer involvement and competition. A key finding of the study considered collaboration and networking of project implementers as a positive attribute of the QED network at its various levels. The experience-sharing component of the network has encouraged the diffusion of knowledge between members.

Within Ethiopia, the network targeted facility-level interaction for experience sharing and learning through competitions and allowing healthcare workers to learn from one another and replicate lessons. These were held biannually on forums that were designed for this purpose. This included using social media channels such as Telegram to expedite the experience-sharing process.

This network is different because it is a network which works on the facilities practically. It has to be learned through idea sharing. The other thing that makes it different is its focus on maternal and child health. Since the aim is that, the idea used in one facility can be replicated in others. If one was working on emergency and the other on maternal cases that would have been a problem. Round1-Local-RHB

In general, the QED network has significantly contributed to coordinating external partners in the area, compelling them to offer technical and financial assistance. Facility observations also revealed that the initiative’s physical renovations have had lasting positive contributions to service delivery. A respondent from a regional bureau highlighted how partner mobilization by the QED network facilitated improvements in NICU room renovations, provision of essential equipment, and neonatal infection prevention, enhancing the quality of care for at least a few consecutive years ahead.

There are hospitals in Addis Ababa including (X) hospital, which have a NICU with higher death rates. Especially in the (X) hospital, death is caused by prematurity-related infections that are increasing. If you have been there, the hospital has a very narrow room and a congested area. So, expansions were carried out and rooms were separated as septic and aseptic. We also strengthened the follow-up of the hospital management system. In addition, we tried to work closely with the ministry to improve problems related to inputs. There are always input problems at the NICU. There were some resource shortages like C-pump and neonatal nasal prologue. That way, we are trying to reduce neonatal death. Interview_ Round 2_Local_RHB

Despite the fact that the QED network contributed to enhanced collaboration, at some levels it was observed that not all stakeholders have the same level of commitment. This was particularly evident in the regional health offices.

Quality work isn’t confined to a quality unit but involves collaboration, problem identification, and effective reporting. Currently, there’s a lack of strong coordination in how the Ministry of Health (MoH) communicates the QED network program, as they simply request one or two participants for meetings, leaving limited room for independent planning and implementation. This highlights a significant coordination issue. Round1-Local-RHB-04

### Rewards

Motivation and rewards play a crucial role in creating and maintaining the momentum of quality culture in an institution. Capacity building and staff motivation through continuous on-job training, monitoring and coaching were some of the approaches applied by the QED network. Allocating sufficient time and intensity of supervision was crucial to observing the motivation of healthcare workers and small steps toward long-lasting improvements.

… For example, I just went to a delivery ward at a given site and they were not working well there. When I go for the second and third follow-ups, they have been doing the same. But later, I saw that they rearranged and innovated the delivery ward. It was so interesting. So, when you motivate the younger staff, they will bring amazing innovations. That was very interesting. So, there are changes. Round 2_national_partner

A respondent from one of the observed facilities also echoed the importance of follow-up to motivate staff as follows.

… Training is something that you forget if you didn’t apply it practically. Therefore, training should be followed by periodic follow-ups. Interview_Local_health centre

Participants suggested that it was essential for staff motivation to be embedded in any intervention aimed at quality improvement. According to the respondents, motivation for behaviour change was an important ingredient for developing a quality culture, not just the provision of materials.

Professionals need to have a mindset for QI; otherwise, it might be difficult even if you invest a lot. When we say quality, people expect special things and think that it requires a big resource. For example, think about what will happen if a health professional provides newborn care with an unclean hand or reuses a glove. These interventions didn’t require resources; it was about washing hands or changing gloves. When you enter the NICU, you are required to change your clothes and shoes and wear goggles, and a head cover. Doing these things doesn’t require resources rather it needs only your motivation. Round 2_national_partner

Even though rewarding high-performing healthcare providers has been critical for maintaining a quality culture, respondents from local facilities repeatedly pointed out implementers’ shortcomings regarding this.

… . the management is not seeing this section as special both for the service and care providers, there should be incentives and refreshments on-work because they are on duty for 24 hrs., and they don’t go for lunch and breakfast. They need incentives. These all have impacted quality. Interview_Local_hospital

### Ownership

This section will emphasize the contributions of the QED network concerning ownership, one of the key attributes of a quality culture. When individuals feel a sense of ownership, they are more likely to take accountability for their actions and decisions. They understand that their contributions directly impact the institution’s performance, which encourages responsible behaviour. One of the manifestations of the QED network’s long-term traces in fostering quality culture was the ownership observed through various innovative ideas by healthcare workers that do not require additional resource mobilization.

Quality is all about what you create, it cannot be installed in you by someone external. For example, regarding a birth hat, for a baby, you advise them that a birth hat is important for a newborn. Then once upon a time, I saw a midwife hand-making a hat from a piece of cloth and giving it to a newborn. This was amazing. She (the midwife) said since they don’t have the supply of hats, she needs to do her own innovation. Interview_Round 2_National _partner

Similarly, another respondent also indicated that quality improvement is more of a mindset thing that can be achieved with individual-level ownership. QED network has supported this by encouraging providers to practice quality with the bare minimum standards.

Keeping equipment clean does not require a considerable resource. Cleaning equipment for 5 minutes is not the same as cleaning for 20 or 30 minutes which is the recommended time; all of these things do not require resources; rather, it is a matter of personal motivation and attitude. Professionals must believe that they can do significant work to improve the quality of care on their own. Interview_ Round 2_National_ Partner

Respondents from the government side also shored up this opinion. Unless the partners’ support is supplemented by facility-level readiness and ownership, the technical and financial support might not be sufficient.

As I said, there are also partners who have built a NICU. They support in such a way, but that is not enough. If you set everything up, it will be useless without a staff member. If you do not have a mechanism to retain the trained staff turnover, and you meet with new people every time the system will lag. Interview_Round2-National-MOH

## Discussion

This study reflected on the efforts of the QED network to cultivate a culture of quality in healthcare in Ethiopia. Five key parameters (leadership, people-centered care, collaboration, rewards, and ownership) showed promise in both the planning and execution of the project. However, challenges have been identified in the effective implementation of the planned activities.

According to a recent study conducted in 115 countries worldwide, an institutional quality culture not only enhances institutional productivity but also fosters the economic complexity of countries [21]. Quality culture in healthcare refers to shared values, beliefs, and behaviors that prioritize patient-centered care and continuous enhancement. It involves fostering accountability, transparency, and collaboration among healthcare providers, continuous service evaluation for improvement, empowering patient involvement in decisions, and ensuring adequate resources and training for quality care. Cultivating a quality culture leads to better patient outcomes, higher staff satisfaction and retention, and enhanced community trust in healthcare institutions [5,19]. An organizational culture that prioritizes patient-centered care and values teamwork and collaboration is likely to lead to better patient outcomes and higher levels of job satisfaction among service providers [22–24].

Quality improvement initiatives often struggle to maintain success as projects phase out and support is terminated [25,26]. The QED network had the potential to realize this gap by following bottom-up approaches that included facility-based interventions focusing on beneficiaries’ satisfaction [19,27]. Characteristics found to be important in institutionalizing a quality culture were the inclusion of learning and experience sharing, coaching and supportive supervision, as well as capacity building, where healthcare professionals acquire skills that influence their behaviour. These characteristics resulted in healthcare professionals living and practising quality as their values rather than simply following an edict from on high [28,29].

The implementation of the QED network had been carefully designed to address both the physical and behavioural aspects of quality improvement. It involved a collaboration of partners providing technical and financial support, including knowledge and skill-related support. Perhaps the most significant aspect of the QED network was the learning platforms that offered a valuable opportunity for skill transfer between facilities. This platform aimed to foster a sense of confidence and empowerment, with the belief that if some low-resource settings can achieve quality care, then others can do it too [8,9,30]. In the case of Ethiopia, it should also be noted that the current Ethiopian healthcare quality and safety strategy plan provided a favorable environment and supportive health system policy through one of its five objectives: to improve the quality culture in health facilities [12].

The study also brings to light certain critical challenges of a structural, behavioural, and skill-related nature that cannot be ignored. These challenges pose formidable hurdles to the network’s effective implementation and realization of its objectives. Moreover, there is a need for further investigation into the degree to which these parameters have been implemented and have influenced the institutional culture.

### Strengths and limitations of this study

This paper illustrated the empirical perspective of fostering a quality culture based on the experiences of the QED network. Based on the lessons learned from this paper, we hope that other comparable networks or initiatives striving to enhance the quality of care will consider taking actions that aim to build and sustain quality as a long-term cultural shift. However, we found that there is a dearth of information in the area, especially from the public health arena, limiting examples from which to draw. Measuring the contribution of the QED network to fostering a quality culture using identified attributes was challenging in the presence of other external factors operating beyond the QED network.

## Conclusion and recommendation

While the QED network in Ethiopia has made important contributions to cultivating a culture of quality care, the full implementation of the five essential attributes – leadership, people-centered interventions, collaboration, rewards, and ownership – remains a work in progress. Despite notable strides in recognizing the importance of these attributes, challenges persist in integrating them fully at different levels of the healthcare system. Ongoing efforts and targeted strategies, including policy refinement and fostering shared responsibility among stakeholders, are essential to ensure that the positive momentum witnessed in the initial phases of the quality improvement initiative translates into a lasting culture of quality care in Ethiopia’s healthcare landscape.
